# Immortalization of Porcine 11β-Hydroxysteroid Dehydrogenase Type 1-Transgenic Liver Cells Using SV40 Large T Antigen

**DOI:** 10.3390/ijms18122625

**Published:** 2017-12-05

**Authors:** Hee Young Kang, Young-Kwon Choi, Yeon Ik Jeong, Kyung-Chul Choi, Sang-Hwan Hyun, Woo-Suk Hwang, Eui-Bae Jeung

**Affiliations:** 1 College of Veterinary Medicine, Chungbuk National University, 1 Chungdae-ro, Seowon-gu, Cheongju, Chungbuk 28644, Korea; heeyoung.kang@daum.net (H.Y.K.); cyk901124@naver.com (Y.-K.C.); kchoi@chungbuk.ac.kr (K.-C.C.); shhyun@cbu.ac.kr (S.-H.H.); 2 Sooam Biotech Research Foundation, 64 Kyunginro, Guro-gu, Seoul 08359, Korea; youniks@sooam.org (Y.I.J.); hwangws@sooam.org (W.-S.H.); 3 Immunotherapy Convergence Research Center, Korea Research Institute of Bioscience and Biotechnology (KRIBB), 125 Gwahak-ro, Yuseong-gu, Daejeon 34141, Korea

**Keywords:** pig, hepatocytes, immortalization, HSD11B1

## Abstract

Cortisol is a steroid hormone essential to the maintenance of homeostasis that is released in response to stress and low blood glucose concentration. Cortisol is converted from cortisone by 11β-hydroxysteroid dehydrogenase type 1 (HSD11B1). It has been reported that too much cortisol or overexpression of HSD11B1 induces obesity and the insulin resistance that accompanies metabolic syndrome in rodent adipose tissue. In our previous study, *HSD11B1*-transgenic (TG) fibroblasts were established, and a porcine model was generated by SCNT using those fibroblasts. Hepatocytes overexpressing HSD11B1 were obtained from livers of this porcine model and cultured in vitro. However, the primary hepatocytes were found to have a short life span or low proliferation rate. To overcome these problems, the SV40 large T antigen was transduced into primary *HSD11B1*-TG hepatocytes, and those cells were immortalized. Immortalized *HSD11B1*-TG hepatocytes showed restored morphology, more rapid proliferation rate, and more expression of HSD11B1 than primary hepatocytes. As well, these cells kept the hepatic characteristics such as gluconeogenic response to cortisone and increased expression of hepatic makers. The immortalized *HSD11B1*-TG hepatocytes may be useful for studying traits and potential therapeutic drugs for treatment of metabolic disorders induced by overexpression of HSD11B1.

## 1. Introduction

The liver plays a major role in metabolism including regulation of glycogen storage, protein synthesis, hormone production and detoxification of various metabolites. The liver primarily consists of hepatocytes, which contribute to the major functions of liver and are a principal target of cortisol. A study of Clinical Endocrinology reported a direct connection between cortisol levels in men and fatty liver disease [1]. According to that study, nonalcoholic fatty liver disease patients had chronic overactivity in the hypothalamo-pituitary-adrenal axis, which led to a subclinical version of Cushing syndrome and the overproduction of cortisol [1].

11β-hydroxysteroid dehydrogenase type 1 (HSD11B1) is a nicotinamide adenine dinucleotide phosphate (NADPH)-dependent enzyme located within the lumen of the endoplasmic reticulum (ER) [2] that is highly expressed in metabolic tissue including that of the liver, adipose tissue, and the central nervous system [3]. HSD11B1 acts as a reductase, converting cortisone into cortisol [4,5]. In the early fasting state, cortisol causes gluconeogenesis or glycogenolysis in liver and muscle, and activates anti-stress and anti-inflammatory pathways. Cortisol also stimulates the activation of glycogen phosphorylase, which is necessary for epinephrine to have an effect on glycogenolysis [6]. *Hsd11b1*-transgenic (TG) mice show abdominal obesity, hyperglycemia, insulin resistance, hyperphagia, hyperleptinemia and increased intra-adipose and portal levels, but not systemic corticosterone levels [7].

In our study, *HSD11B1*-TG male pigs were generated as a disease model for metabolic syndrome by somatic cell nuclear transfer (SCNT) using recloned fibroblasts. Recloned fibroblasts were primary cultured cells obtained from the first male pig generated via SCNT using transgenic porcine fibroblasts that had been established in our laboratory in 2013 [7]. Since HSD11B1 has been associated with several metabolic disorders, it has been investigated as a novel target for potential therapeutic drugs [8]. Thus, *HSD11B1*-TG hepatocytes will be useful for studying metabolic diseases such as hyperglycemia and fatty liver disease. However, establishment of a *HSD11B1*-TG hepatic cell line has not been reported to date. In this study, hepatocytes obtained from *HSD11B1*-TG male pigs were cultured. These primary hepatocytes could be maintained for several days and until 10 passages; however, their proliferation rate decreases, and eventually stops with morphological change [9].

This blockage of cell proliferation can be overcome by SV40 oncogene large T antigen (SV40LT), which inactivates both p53 and retinoblastoma [10]. Cell immortalization is accomplished through a two-stage process. Specifically, cells expressing SV40LT escape senescence but continue to lose telomeric repeats during their extended life span. Eventually, terminal telomere shortening causes the cells to reach a second non-proliferative stage termed “crisis” [11]. Escape from crisis is a very rare event accompanied by the reactivation of telomerase [12]. 

In this study, we introduced *SV40LT* into primary *HSD11B1*-TG (Pri11βTG) hepatocytes to establish immortalized cells in vitro. Since *SV40LT*-*HSD11B1*-TG (SV11βTG) hepatocytes contain oncogenes, these cells proliferate well under in vitro conditions. We then investigated whether the SV11βTG hepatic cell line kept the original characteristics of hepatocytes and if the morphology of immortalized hepatocytes was recovered.

## 2. Results

### 2.1. Confirmation of Altered Morphology and Genomic Hybridization of SV40LT in SV11βTG Hepatocytes 

**Figure 1:**
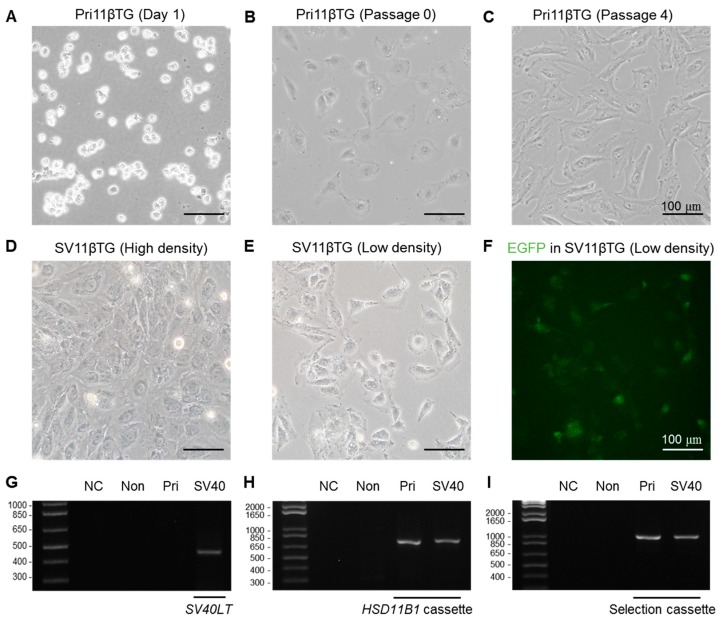
Morphological change and PCR-based confirmation of genomic integration in immortalized SV11βTG hepatocytes. (**A**–**C**) Accordance with the progression of passages (one day after cell isolation (**A**), the zero passage (**B**), and the fourth passage (**C**)), morphology of Pri11βTG hepatocytes became longer and fibroblast-like, and the size of hepatocytes increased; (**D**–**F**) Morphology and size of the SV11βTG hepatocytes, which were immortalized by SV40 large T antigen, recovered similar to Pri11βTG ones at the first passage in high density (**D**) and low density (**E**); EGFP expression (**F**) was confirmed in 11βTG hepatocytes due to the selection cassette including *EGFP* and neomycin resistance gene (*Neo^r^*) [7] (Scale bar, 100 µm); (**G**–**I**) Chromosomal insertion was confirmed by PCR using primers flanking the target cDNA in *SV40LT* sequences (**G**) for immortalization, in *HSD11B1* expression cassette (**H**) and in selection cassette (**I**). (NC, negative control without template; Non, non-transgenic hepatocytes; Pri, Pri11βTG hepatocytes; SV40, SV11βTG hepatocytes; *SV40LT*, SV40 large T antigen).

Pri11βTG hepatocytes were isolated from the liver of *HSD11B1*-TG male pigs and immortalized through *SV40LT* transduction. One day after the cell isolation, the hepatocyte was weakly adherent and showed a small round shape (Figure 1A). After 4 days, the cells were strongly adhered and started to grow (Figure 1B). Morphology of Pri11βTG hepatocytes became larger and longer as the passage progressed (Figure 1C). On the other hand, SV11βTG hepatocytes became smaller as passage progressed, and were similar to primary hepatocytes with passage 0–2 (Figure 1B,E). Immortalized SV11βTG hepatocytes have recovered hepatic morphology and size at approximately passage 35 (Figure 1D–F). PCR was then performed using primer sets specific for the vector to confirm the chromosomal integration of the targeting vector (Table 1). The *SV40LT* gene was only observed in genomic DNA of immortalized SV11βTG hepatocytes (Figure 1G). The *HSD11B1* cassette and selection cassette were confirmed in genomic DNA of both Pri11βTG and SV11βTG hepatocytes (Figure 1H,I). Both Pri11βTG and SV11βTG hepatocytes express EGFP due to the selection cassette with the *EGFP* gene (Figure 1F).

### 2.2. Expression of SV40LT mRNA and Increase of Cell Proliferation Rate in SV11βTG Hepatocytes

**Figure 2:**
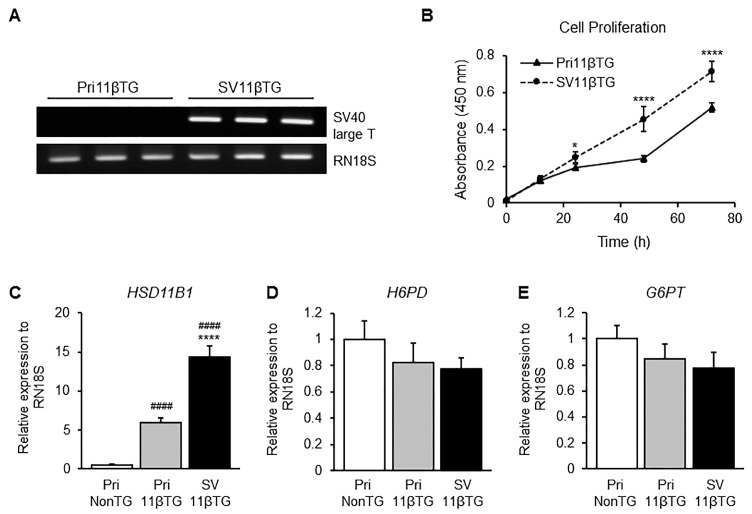
Cell-proliferation rate in SV11βTG hepatocytes expressing *SV40LT* and effect of HSD11B1 overexpression on *H6PD* and *G6PT* mRNA in hepatic G6PT-H6PD-HSD11B1 triad system. (**A**) The mRNA expression of *SV4LT* was observed in immortalized SV11βTG hepatocytes by PCR; (**B**) The proliferation rate of Pri11βTG (▲) and SV11βTG (●) hepatocytes for three days was measured by CCK-8 assay. The rate was significantly elevated in SV11βTG hepatocytes. Each group was prepared to half-dozen wells, and this experiment was performed twice; (**C**) Expression of *HSD11B1* was highly increased; (**D**,**E**) Expression of *H6PD* (**D**) and *G6PT* (**E**) was insignificantly changed by transduction of *HSD11B1* gene and immortalization using SV40LT antigen. Relative expression levels of mRNA were investigated by qPCR and normalized to *RN18S*. These experiments were performed in triplicate and independently three times. Number signs and asterisks indicate significant difference comparing to PriNonTG (^####^ *p* < 0.0001) and Pri11βTG (* *p* < 0.05, **** *p* < 0.0001) hepatocytes, respectively. White, gray and black bars represent PriNonTG, Pri11βTG and SV11βTG hepatocytes, respectively.

To confirm that mRNA of *SV40LT* is stably expressed, reverse transcription using total RNA and PCR was performed. Expression of *SV40LT* mRNA was only observed in SV11βTG hepatocytes (Figure 2A). After immortalization by *SV40LT*, SV11βTG hepatocytes grew more rapidly and showed greater proliferation than Pri11βTG hepatocytes. A CCK-8 assay was carried out to assess the difference in proliferation rate (Figure 2B). The line slope for absorbance to time (h) is 0.0062 in Pri11βTG hepatocytes, while it is 0.0095 in SV11βTG hepatocytes. The proliferation rates of SV11βTG hepatocytes were significantly (1.53 times) faster than those of Pri11βTG hepatocytes

### 2.3. Overexpression of HSD11B1 mRNA but No Significant Changes of H6PD and G6PT mRNA in SV11βTG Hepatocytes

It has been reported that cortisol production is catalyzed via the triad system, which consists of glucose-6-phosphate transporter (G6PT or SLC37A4), hexose-6-phosphate dehydrogenase (H6PD) and HSD11B1 in ER of hepatocytes, and that the reductase activity of HSD11B1 was activated by NADPH generated via H6PD [2,14]. *HSD11B1* showed more expression in SV11βTG than Pri11βTG hepatocytes (Figure 2C). Although *HSD11B1* increased significantly, the expression level of *H6PD* and *G6PT* did not change significantly (Figure 2D,E). These results indicate that the overexpression of *HSD11B1* does not directly affect the expression of *H6PD* and *G6PT*. 

### 2.4. Verification of Functional Hepatocytes through Expression of Gluconeogenic Markers

**Figure 3:**
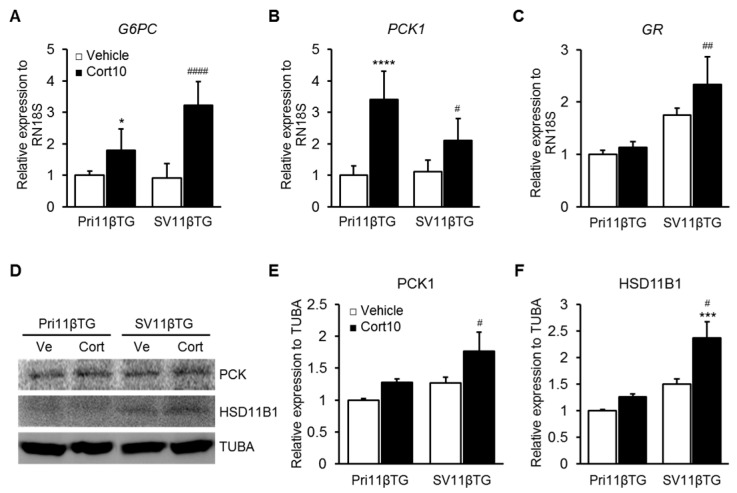
Induction of gluconeogenic markers by cortisone treatment. (**A**,**B**). The mRNA levels of gluconeogenesis-related genes such as *G6PC* (**A**) and *PCK1* (**B**) were significantly increased in cortisone-treated group of both Pri11βTG and SV11βTG hepatocytes; (**C**) The mRNA level of *GR* binding to cortisol was more increased in SV11βTG hepatocytes than Pri11βTG ones. Relative level of these mRNAs was normalized to *RN18S*; (**D**) protein level of PCK1 and HSD11B1 was examined by Western blotting method; (**E**,**F**) Relative expression of these protein was quantified by ImageJ program and normalized to α-tubulin. Both PCK1 and HSD11B1 proteins levels meaningfully increased in cortisone-treated condition of SV11βTG hepatocytes. Asterisks and number signs respectively mean significant increase comparing to vehicle-treated group of Pri11βTG (* *p* < 0.05, *** *p* < 0.001 and **** *p* < 0.0001) and SV11βTG (^#^ *p* < 0.05, ^##^ *p* < 0.01 and ^####^ *p* < 0.0001) hepatocytes. All experiments were performed independently at least three times. White bars represent vehicle and black bars represent cortisone-treated group. (Ve, vehicle; Cort, cortisone)

Cortisol converted by HSD11B1 facilitates hepatic gluconeogenesis. To identify the effects of HSD11B1 overexpression on gluconeogenic genes including glucose-6 phosphatase catalytic subunit (*G6PC*, Figure 3A) and phosphoenolpyruvate carboxykinase 1 (*PCK1*, Figure 3B), we measured mRNA levels by real-time PCR and PCK1 protein levels by Western blotting (Figure 3D). The mRNA expression of *G6PC* and *PCK1* increased significantly in response to treatment with cortisone, the substrate of HSD11B1, in both Pri11βTG and SV11βTG hepatocytes (Figure 3A,B), while no difference in expression of either gene was observed in dimethyl sulfoxide (DMSO) treated cells, as vehicle control. Similar to the mRNA level, expression of HSD11B1 protein in SV11βTG hepatocytes was greater than in Pri11βTG hepatocytes (Figure 3D,F). Moreover, expression of PCK1 protein increased slightly under cortisone treatment (Figure 3D,E). Also, we examined whether overexpression of HSD11B1 induces the expression of glucocorticoid receptor (*GR*, Figure 3C), which binds to cortisol or other glucocorticoids and acts as a effector in the cortisol-mediated response [15,16]. The level of *GR* mRNA was meaningfully increased in cortisone-treated condition of SV11βTG hepatocytes, similar to increasing pattern of HSD11B1 mRNA and protein. These findings suggest that transgenic HSD11B1 acts as a functional reductase, is not affected by the immortalization process, and that SV11βTG hepatocytes retain the traits of Pri11βTG hepatocytes.

### 2.5. Confirmation of Hepatic Character Using Liver Markers

**Figure 4:**
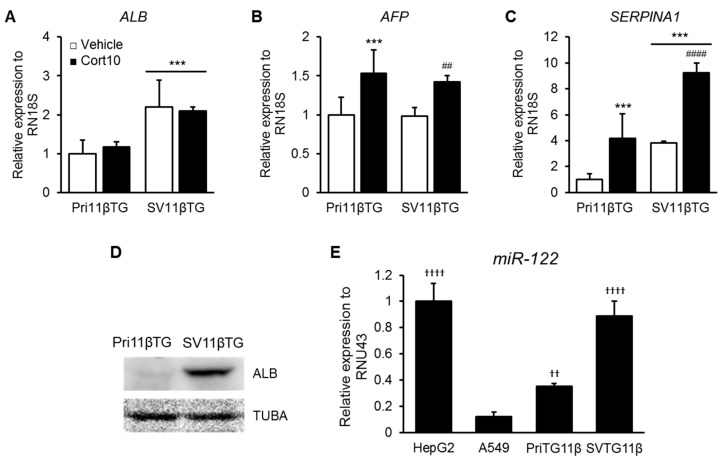
Identification of hepatic markers. (**A**–**C**) Whether Pri11βTG and SV11βTG hepatocytes keep hepatic traits was examined by qPCR using primer to hepatic markers including *ALB* (**A**), *AFP* (**B**), and *SERPINA1* (**C**), (**A**,**C**); The mRNA levels of *ALB* and *SERPINA1* was significantly elevated in SV11βTG hepatocytes; (**B**,**C**) *AFP* and *SERPINA1* mRNA levels were meaningfully increased in cortisone-treated group. Asterisks and number signs mean significant increase comparing to vehicle-treated group of Pri11βTG (*** *p* < 0.001) and SV11βTG (^##^ *p* < 0.01 and ^####^ *p* < 0.0001) hepatocytes respectively. Relative mRNA level was normalized to *RN18S*. These experiments were repeated independently three times. White bars represent vehicle and black bars represent cortisone-treated group; (**D**,**E**) Hepatic markers including ALB protein (**D**) and *miR-122* (**E**) was identified and increased in SV11βTG hepatocytes. A549, a lung cancer cell line, was used as a negative control. Daggers indicate significant difference comparing to A549 cell line ((^††^ *p* < 0.01 and ^††††^ *p* < 0.0001). The relative miRNA level was normalized to *RNU43*. These experiments were repeated twice in triplicate.

To identify the hepatic traits of cultured hepatocytes, we measured mRNA levels of liver specific genes such as albumin (*ALB*, Figure 4A), α-fetoprotein (*AFP*, Figure 4B) and serpin peptidase inhibitor α1-antitrypsin member 1 (*SERPINA1*, Figure 4C), and confirmed expression of ALB protein (Figure 4D). Expression of ALB protein as well as *ALB* and *SERPINA1* mRNA was identified in SV11βTG cells and, increased significantly than in Pri11βTG cells (Figure 4A,C,D). It means that immortalized SV11βTG cells should be hepatocytes. Moreover, mRNA levels of *AFP* and *SERPINA1* were up-regulated in both Pri11βTG and SV11βTG hepatocytes by treatment with 10 μM cortisone (Figure 4B,C). The liver-enriched microRNA-122 (*miR-122*) level in SV11βTG hepatocytes was similar to one in HepG2, and was more expressed than in Pri11βTG hepatocytes (Figure 4E). These results suggest that SV11βTG hepatocytes recover, or retain the characteristics of hepatocytes and are useful for the functional hepatocytes.

## 3. Discussion

Since pigs are mono-gastric omnivores and have anatomical and physiological characteristics highly comparable to those of humans, porcine models are suitable for investigating and understanding the biological pathways related to human metabolic diseases [17]. In this study, we established immortalized porcine *HSD11B1*-TG (SV11βTG) hepatocytes, which are useful for investigation of metabolic disorders, through transduction of SV40LT antigen. In these immortalized cells, we confirmed integration of the *HSD11B1* gene cassette, selection cassette with the *Neo^r^* and *GFP* genes, and the *SV40LT* gene. 

HSD11B1 mRNA and protein were overexpressed in SV11βTG hepatocytes; hence, the expression of *G6PT* and *H6PD*, which act as triads with HSD11B1 in ER, has no significant effect on mRNA level. These results may have been attributed to different basal expression levels of the three genes. The expression level of H6PD was 50–60 times higher than HSD11B1 and that of G6PT was 7–9 times higher than HSD11B1. G6PT enzyme is a transmembrane protein consisting of three transporting subunits (G6PT1, G6PT2 and G6PT3) that provides a selective channel and transports glucose-6-phosphate produced in the terminal reactions of both glycogenolysis and gluconeogenesis from the cytosol into the lumen of the ER. During this process, H6PD produces the cofactor NADPH for HSD11B1 activation [2]. Activated HSD11B1 converts inactive cortisone into active cortisol, which increases the glucose level in blood through gluconeogenesis and is involved in the metabolism of fat, protein, and carbohydrates [6]. Increased level of *GR* mRNA may indicate the increase in cortisol converted by HSD11B1 in SV11βTG hepatocytes.

In a study using *db*/*db* mice, it has been reported that increased expression of GR and HSD11B1 in hepatocytes is an important component in the development of type 2 diabetes [18]. Also, hepatic GR deficiency by delivering an adenovirus expressing GR-specific shRNA improves hepatic steatosis in livers of *db*/*db* mice [19]. The GR has been characterized as a crucial regulator of glucose homeostasis [19], belongs to nuclear receptor subfamily 3, group C, member1 (NR3C1) and binds to cortisol [14]. Complex consisting of GR and cortisol regulates transcription of genes related to the development, metabolism and immune response [16]. In this study, this complex has induced the expression of gluconeogenic and downstream genes such as *G6PC* and *PC*K*1*. However, overexpression of HSD11B1 did not induce the increase of *G6PT* and *H6PD* expression in the hepatic G6PT-H6PD-HSD11B1 triad system. 

To verify the hepatic traits of SV11βTG hepatocytes, we examined the expression of liver markers including ALB, *AFP*, *SERPINA1*, and *miR-122*. Most of these markers increased in immortalized hepatocytes. ALB protein is made specifically in the liver [20]. Primary cultured cells lost the inherent properties and morphology of original tissue in accordance with the progression of passages. Increased expression of ALB protein in SV11βTG hepatocytes indicates that SV11βTG hepatocytes had recovered from the loss of tissue specificity. It is also supported by the fact that *miR-122*, which is necessary for the regulation of liver development, differentiation and metabolic function [21], has been further increased in SV11βTG hepatocytes than in Pri11βTG hepatocytes. The temporal regulation of *miR-122* has been reported to promote hepatobiliary segregation with the acquisition and maintenance of hepatocyte phenotype, and to be involved in the regulation of cholesterol and fatty acid metabolism [21]. In some murine studies, genetic deletion of *miR-122* showed severe dysfunction on lipid metabolism, and micro-steatosis, fibrosis and inflammation in the liver [22,23]. 

AFP was relatively highly expressed in the fetal liver [24], and AFP level in the serum decreased in the weeks and months after birth [25]. As shown in Figure 4, *AFP* level did not differ between Pri11βTG and SV11βTG, but increased in response to treatment with cortisone. Some studies have reported that temporary treatment of cortisol or dexamethasone in hepatic tumor reduces AFP [26,27]. However, in a 10-year-old boy with pancreatic acinar cell tumor, Cushing’s syndrome, which was diagnosed with high serum adrenocorticotropic hormone and cortisol levels, was identified with the tumor, and excessive AFP level in serum was observed [28]. This clinical result differs from other studies in that it is continuously exposed to cortisol, and HSD11B1 is expected to be highly expressed in the patient, considering the increase of AFP expression in cortisone treatment in our 11βTG hepatocytes. Also, an elevated AFP concentration in the serum of adults was primarily observed in patients with hepatocellular carcinoma, chronic hepatitis, and acute liver failure [29]. Considering the influence of cortisol on hepatic metabolism, these findings suggest that cortisol can affect AFP levels during metabolic stress responses. 

Since SERPINA1 is expressed in the liver [30], it was used to confirm hepatic traits. SERPINA1 is one of the most abundant serine protease inhibitors belonging to the serpin superfamily and is known by other names such as α1-antitrypsin (A1AT) and α1 protease inhibitor (A1PI) because of its ability to inhibit various proteases [31]. SERPINA1 is capable of increasing greatly upon acute inflammation. SERPINA1 plays a key role in protection of tissues from protease of inflammatory cells, especially neutrophil elastase [32]. Deficiency and mutation of SERPINA1 can cause pulmonary emphysema via uncontrolled elastase activity and liver cirrhosis through accumulation of misfolded proteins and impaired secretion [33]. Therefore, the use of three SERPINA1 products obtained from human plasma, Prolastin, Zemaira, and Aralast, was approved by the United States Food and Drug Administration [34]. It has been reported that treatment with SERPINA1 products significantly reduced neutrophils, TNF-α, and neutrophil chemokine KC (CXCL1) [35]. Hence, it was reported that cortisol, a steroid hormone, suppresses the immune system by preventing production of IL12, IFNγ, IFNα and TNFα via antigen presenting cells and T helper cells [36]. As shown in Figure 4, the levels of *SERPINA1* increased in immortalized SV11βTG hepatocytes and were significantly elevated in response to treatment with cortisone. These findings suggest that cortisol increases the expression of SERPINA1, and hepatic traits in SV11βTG hepatocytes had been restored. 

In conclusion, we used the SV40LT system for immortalization. Although human liver sinusoidal endothelial cells have previously been immortalized using the h*TERT* gene [37], previous studies have never reported immortalization of porcine *HSD11B1*-TG hepatocytes by introduction of *SV40LT* in vitro. Furthermore, these cells have a tendency to maintain their original characteristics, such as response to cortisone and gluconeogenesis. Overexpression or dysregulation of HSD11B1 has been associated with metabolic disorders eliciting abdominal obesity, hyperglycemia, hyperphagia, hyperleptinemia, and insulin resistance. Recently, HSD11B1 has been investigated as a novel target of potential therapeutic drugs for metabolic syndrome, including Cushing syndrome. Thus, our immortalized SV11βTG hepatocytes may be useful for investigating traits and potential therapeutic drugs for metabolic disorders.

## 4. Materials and Methods 

### 4.1. Animals and Primary Porcine Hepatocytes Culture

The pig experiments were performed in strict accordance with the recommendations in the Guide for the Care and Use of Laboratory Animals of the National Veterinary and Quarantine Service, and this study was approved by the animal ethics committee of Sooam Biotech Research Foundation (P-15-01) and the Committee on the Ethics of Animal Experiments of the Chungbuk National University (CBNUA-871-15-01). All efforts were made to minimize animal suffering.

### 4.2. Isolation of Primary Porcine Hepatocytes 

Liver was removed from Yucatan male pig, washed in cold PBS, chopped on cold petri dish, and dispersed with 0.05% trypsin at 37 °C for 15 min. Supernatant was mixed to the same volume of basal medium (Dulbecco Modified Eagle Medium (DMEM) with 25 mM glucose, 3.7 g/L sodium bicarbonate (pH 7.4), 10% fetal bovine serum (FBS), 100 U/mL penicillin, 100 μg/mL streptomycin, and 5 μg/mL plasmocin) and centrifuged (1200 rpm for 5 min). The pellet was resuspended in 4 mL of basal medium and layered over a percoll gradient in phosphate-buffered saline (PBS) containing 70 to 5% percoll (vol/vol). After centrifugation for 20 min at 3600 rpm, the middle layer among three regions was collected. The collected cell was cultured in basal media and in a humidified 5% CO_2_ atmosphere at 37 °C. 

### 4.3. Cell Treatments

Pri11βTG or SV11βTG hepatocytes (1 × 10^5^ cells per well) were attached for one day, then starved in starvation medium (DMEM with 5 mM glucose without FBS and phenol-red) for one day before being treated with 0.1% DMSO or 10 μM cortisone in starvation medium containing 10% charcoal-dextran treated FBS for two days. 

### 4.4. Immortalization of Primary Hepatocytes

To introduce *SV40LT* into primary cell, retroviral vector pLXIN-SV40T was stably transfected into the recombinant retrovirus packaging cell line, PT67 cells (Takara, Shiga, Japan). Retrovirus-containing medium was collected, and transferred onto Pri11βTG hepatocytes [38]. 

### 4.5. Genomic DNA Extraction and Confirmation of SV40T Integration

Genomic DNA was isolated with a G-DEX™ IIc Genomic DNA Extraction kit (iNtRON, Gyeonggi-do, South Korea). 100 ng of genomic DNA was amplified in a 20 μL PCR reaction containing 1 U i-Start Taq polymerase (iNtRON), 2 mM dNTPs (Takara) and 10 pmol of each specific primer listed in Table 1. Amplicons were separated on 1% or 1.5% agarose gel, stained with ethidium bromide, photographed under UV illumination, and scanned using GelDoc EQ (Bio-Rad, Hercules, CA, USA). 

### 4.6. Cell Counting Kit-8 (CCK-8) Assay

The number of cells on 96 well-plate was seeded to 4 × 10^3^ cells per well. The cell was maintained in basal medium and cultured for 10, 28, 48, and 72 h. These cells were incubated with CCK-8 solution (Dojindo, Rockville, MD, USA) for 2 h. Absorbance of each well was measured at 450 nm. 

### 4.7. RNA Isolation and Gene Expression Analysis by RT-PCR and qPCR

Total RNA was extracted using TRI Reagent (Thermo Fisher Scientific, Carlsbad, CA, USA) according to the manufacturer’s instructions. cDNA was synthesized using *MMLV* reverse transcriptase (Thermo Fisher Scientific) and random primers (TaKaRa; 9-mers) or stem-loop RT primer (*miR-122*, GTTGGCTCTGGTGCAGGGTCCGAG GTATTCGCACCAGAGCCAACAACGCC [39]; RNU43, GTTGGCTCTGGTGCAGGGTCCGAGGT ATTCGCACCAGAGCCAACAATCAG [13]). Prime Q-master mix (GeNet Bio, Daejeon, South Korea) was used for quantitative PCR (qPCR). Primer sequences of target genes are described in the Table 1. Relative expression mRNA and miRNA was calculated using the equation R = 2^−Δ(Δ*C*tTarget – Δ*C*tInternal control)^ normalized by *RN18S* and *RNU43*, respectively. RNU43, which called the small-nucleolar RNA, C/D Box 43 (SNORD43), belongs to the large C/D box family and function in 2′-*O*-ribose methylation in ribosome biogenesis.

### 4.8. Protein Expression Analysis by Western Blotting

Lysates were extracted in RIPA buffer (50 mM Tris, pH 7.4, 150 mM sodium chloride (NaCl), 1% Triton X-100, 0.5% sodium deoxycholate, 1 mM EDTA and 1 mM PMSF) containing protease inhibitor cocktail (Roche, Basel, Switzerland) by vortex. 70 μg of denatured protein was used in Western blotting and treated with the primary antibodies (HSD11B1 (Novusbio, Littleton, CO, USA; NBP1-69644, 1:1000), PCK1 (Cayman, Ann Arbor, MI, USA; 10004943, 1:1000), ALB (Dako, Glostrup, Denmark; A0001, 1:1000) and α-tubulin (Cell signaling, Danvers, MA, USA; #2144, 1:1000) and horseradish peroxidase (HRP)-conjugated rabbit secondary antibodies. After washing, the blots were exposed to enhanced chemiluminescence (ECL) reagent (Santa Cruz Biotech, Dallas, TX, USA) and then were developed.

### 4.9. Statistical Analysis

Data are presented as means ± standard deviation (SD) and were analyzed by one-way or two-way ANOVA (GraphPad Prism Software, Inc., San Diego, CA, USA). *p* < 0.05 was considered to indicate a statistically significant difference.

## Figures and Tables

**Table 1:** Primer sequences used in polymerase chain reaction (PCR).

**Primer**	**Genebank ID**		**Sequences (5′ to 3′)**
HSD11B1	NM_214248	F	CAACGTGTCAATCACGCTCT
		R	TTCCTGGATTTTCCAACAGG
H6PD	XM_005674044	F	CAGGCTGGAGGAGTTCTTTG
		R	AGGTCTCGCGGATGTTCTT
G6PT	GU207843	F	CTGTCTCCTGCTCATCCACA
		R	AGAACTGGCCCCAGTCAGTA
G6PC	EU717834	F	AAGTTGTTGCTGGGGTCTTG
		R	CCTTCGCTTGGCTTTCTCTA
PCK1	NM_001123158	F	GAGCACAAGGGCAAAGTGATTAT
		R	GAGCCAGTGGGCCAGGTATT
GR	NM_001008481	F	AGCATGCCGCTACAGAAAGT
		R	GACTTCCAGCAGTGACACCA
ALB	NM_001005208	F	TGTCTTCCTGGGCACGTTTT
		R	TAGGCTCATCCACAAGAGGC
AFP	NM_214317	F	TGCTTTCAAACAAAGGCAGCA
		R	ACTCCAGCACGTTTCCTCTG
SERPINA1	NM_214395	F	ACCCAAGTTCTGCCAATCTACA
		R	GTGGCCTCTGTCCCTTTCTC
RN18S	NR_046261	F	CGCGGTTCTATTTTGTTGGT
		R	AGTCGGCATCGTTTATGGTC
**Primers to Confirm Genomic Integration**			
HSD11B1 cassette		F	CCATGATAATAAGCCTGCTCTACTCCA
		R	GGAAGTCATGAAGGCCTGGGTGATG
Selection cassette		F	CATGAAGCAGCACGACTTCT
		R	CCTAGGAATGCTCGTCAAGA
SV40LT	NC_001669	F	CTGACTTTGGAGGCTTCTGG
		R	GGAAAGTCCTTGGGGTCTTC
**Primers to Detect miR-122 and RNU43**			
miR-122		F	TGGAGTGTGACAATGGTGTTTG
		R	AACGCCATTATCACACTAAATA
RNU43 [13]		F	GTGAACTTATTGACGGGCG
		R	GTGCAGGGTCCGAGGT

## Abbreviations

HSD11B1	11β-hydroxysteroid dehydrogenase type 1
TG	Transgenic
ER	Endoplasmic reticulum
SCNT	Somatic cell nuclear transfer
SV40LT	SV40 oncogene large T antigen
Pri11βTG	primary HSD11B1-TG
SV11βTG	SV40LT HSD11B1-TG
G6PT	Glucose-6-phosphate transporter
H6PD	Hexose-6-phosphate dehydrogenase
G6PC	Glucose-6 phosphatase catalytic subunit
PCK1	Phosphoenolpyruvate carboxykinase 1
GR	Glucocorticoid receptor
ALB	Albumin
AFP	α-fetoprotein
SERPINA1	Serpin peptidase inhibitor α1-antitrypsin member 1
